# Thoracic ultrasound finding interstitial lung disease in SLE: a diagnostic test accuracy study

**DOI:** 10.1093/rap/rkag126

**Published:** 2026-10-14

**Authors:** Henrik Zachar Langkilde, Bjørk K Sofíudóttir, Jesper Rømhild Davidsen, Stefan Markus Walbom Harders, Stefan Møller Luef, Susan Due Kay, Elisabet Svenungsson, Sille Fløjborg, Robin Christensen, Anne Voss

**Affiliations:** Research Unit of Rheumatology, Department of Clinical Research, University of Southern Denmark, Odense University Hospital, Odense, Denmark; Department of Rheumatology, Midt og Vestsjællands Sygehus, Slagelse, Denmark; Research Unit of Rheumatology, Department of Clinical Research, University of Southern Denmark, Odense University Hospital, Odense, Denmark; Odense Respiratory Research Unit (ODIN), Department of Clinical Research, University of Southern Denmark, Odense University Hospital, Odense, Denmark; South Danish Center for Interstitial Lung Diseases (SCILS) and Pulmo-Rheuma Frontline Center (PURE), Department of Respiratory Medicine, Odense University Hospital, Odense, Denmark; Research Unit of Department of Radiology, University of Southern Denmark, Odense University Hospital, Odense, Denmark; Odense Respiratory Research Unit (ODIN), Department of Clinical Research, University of Southern Denmark, Odense University Hospital, Odense, Denmark; Research Unit of Rheumatology, Department of Clinical Research, University of Southern Denmark, Odense University Hospital, Odense, Denmark; Division of Rheumatology, Department of Medicine Solna, Karolinska Institutet, Karolinska University Hospital, Stockholm, Sweden; Systemic Lupus Erythematosus Patient Research Partner, Department of Rheumatology, Odense University Hospital, Odense, Denmark; Research Unit of Rheumatology, Department of Clinical Research, University of Southern Denmark, Odense University Hospital, Odense, Denmark; Section for Biostatistics and Evidence-Based Research, The Parker Institute, Copenhagen University Hospital, Bispebjerg and Frederiksberg, Copenhagen, Denmark; Research Unit of Rheumatology, Department of Clinical Research, University of Southern Denmark, Odense University Hospital, Odense, Denmark

**Keywords:** SLE, interstitial lung disease, thoracic ultrasound

## Abstract

**Objectives:**

Interstitial lung diseases (ILDs) are frequent and clinically important in SLE but diagnosis of ILD can be challenging. Thoracic ultrasound (TUS) shows promise in detecting ILD, hence, we aimed to evaluate the utility of TUS to identify ILD in SLE.

**Methods:**

Patients fulfilling the EULAR/ACR 2019 SLE classification criteria were invited. Participants were assessed for ILD by means of chest high-resolution computed tomography scans and pulmonary function tests and diagnosed at a multidisciplinary discussion, according to reference standards, independent of TUS. TUS evaluated the presence of B-lines and pleural abnormalities, blinded to status of ILD. The ability of B-lines to identify ILD using a prespecified threshold of ≥10 B-lines was analysed using diagnostic test accuracy measures. Further, we explored the diagnostic performance of B-lines and pleural abnormalities to identify ILD across various scenarios.

**Results:**

Among 185 patients with SLE, ILD were present in 22 (12%). With the predefined threshold of ≥10 B-lines, we found a modest diagnostic test accuracy as 14 of 22 ILD cases were classified as abnormal TUS, with diagnostic odds ratio of 10.7 (3.2–32.3), sensitivity of 64% (41–83%) and negative predictive value 97% (90–98%). In exploratory analyses, excluding participants with certain characteristics possibly affecting the TUS from the analyses improved the performance of TUS and pleural abnormalities were associated with ILD.

**Conclusion:**

In SLE, TUS focusing on the presence of B-lines demonstrated modest but statistically significant diagnostic test accuracy for detecting ILD. Pleural abnormalities were significantly associated to ILD.

Key messages

- In diagnostic test accuracy analyses thoracic ultrasound (TUS) focusing on B-lines was significantly associated with interstitial lung diseases (ILD) in SLE with a modest performance, but a high negative predictive value.
- TUS focusing on an abnormal pleura was significantly associated with ILD in SLE in exploratory analyses.
- Exploratory analyses indicated that TUS focusing on B-lines might fail to identify certain subtypes of ILD in SLE and ILD in patients with body mass indices >30 kg/m^2^.

## Introduction

SLE is an autoimmune disease characterized by the presence of specific autoantibodies and involvement of multiple organs [1]. Pulmonary diseases are estimated to affect 50% of patients with SLE [2–4], where they are associated with increased mortality [5, 6] and decreased quality of life [7, 8]. Pulmonary diseases cover different pulmonary manifestations including interstitial lung disease (ILD) [2–4, 9], and SLE-related ILD (SLE-ILD) comprises radiological subtypes including non-specific interstitial pneumonia (NSIP), usual interstitial pneumonia (UIP), organizing pneumonia (OP) and lymphocytic interstitial pneumonia (LIP) and is associated with increased mortality [5, 9–11]. SLE-ILD is included in the recent 2025 European Respiratory Society (ERS)/European Alliance of Associations for Rheumatology (EULAR) guidelines on connective tissue disease-associated ILD (CTD-ILD), but because of lack of evidence, no recommendations are stated [12].

The prevalence of SLE-ILD is reported to be 11% in a recent systematic review, increasing to 18% when diagnosis is based on chest high-resolution scans (HRCTs). The systematic review demonstrated higher prevalences of ILD in clinical studies as compared with registry studies indicating the importance of dedicated investigations of the lungs [11]. Similarly, a study reported a prevalence of SLE-ILD 4% based on registry data increasing to 16% after adding tests targeting ILD [13]. In prior studies different methods have been used to detect SLE-ILD, such as chest X-rays (CXR) and pulmonary function tests (PFT), however, adding these examinations do not seem to identify ILD as efficiently as HRCTs [9, 14, 15]. HRCT is the radiological gold standard for identifying ILD and central in obtaining a multidisciplinary discussion (MDD) diagnosis of ILD, which is the reference standard [16, 17]. However, the number of HRCTs should be limited because of patient exposure to radiation [18], possible capacity constraints and high costs of HRCT scanning [19]. Therefore, easily accessible and radiation-free tools to identify ILD in SLE are warranted.

In other systemic autoimmune rheumatic diseases (SARD) protocolized thoracic ultrasound (TUS) has shown to be beneficial in detecting findings indicative of underlying ILD [20–26], but it has only been sparsely described in SLE-ILD [20, 27]. Prior studies have used different thresholds, but a diagnostic test accuracy (DTA) study investigating TUS and ILD in patients with SARD that used a 14 zones protocol at our centre used a threshold of ≥10 B-lines, which performed well among patients with rheumatoid arthritis [28]. One study described abnormal TUS findings among 75% of patients with SLE, however, the association between TUS and ILD was not reported [29]. SLE-ILD may present differently from other SARD-related ILD and often present with air-containing cysts in the lung tissue [30], which can be particularly difficult to detect by TUS [31].

In the present study, we investigated the utility of TUS in patients with SLE in order to: (1) evaluate the DTA of B-lines to identify SLE-ILD; (2) explore the utility of pleural abnormalities for identifying SLE-ILD.

## Methods

### Summary of project methods

The study is a population-based, cross-sectional, DTA and exploratory study. At a visit day, participants underwent TUS, clinical examination, PFT and HRCT. TUS was performed in the out-patient clinic and pseudonymized TUS video clips were stored. TUS clips were scored blinded to other clinical information. HRCTs were assessed by a radiologist blinded to the other measures, and ILD was diagnosed and categorized to subtypes at a MDD blinded to the TUS findings.

### Ethics, registration, data handling and reporting

The study was registered at clinicaltrials.gov (NCT06087523) prior to the first study visit, approved by the regional committee on health research for Southern Denmark (S-20210164) and data protection agency (23/22079). Data were stored in a Research Electronic Data Capture (REDCap) database. The study is reported according to the Standard for Reporting Diagnostic Accuracy (STARD) statement [32] (checklist in Supplementary Data S1, available at *Rheumatology Advances in Practice* online). A patient partner reviewed the study protocol, the statistical analysis plan (SAP) and the final manuscript.

### Study population and inclusion

Participants were identified by a systematic search in our electronic patient record system covering all hospitals across Region of Southern Denmark (catchment population of 1.23 million inhabitants). The search included visits related to SLE or SLE-associated diseases (ICD10 codes DL184A, DL930, DL930A, DM321, DM328 and DM329) between 1 July 2021 and 30 June 2023. According to the predefined protocol, we included participants diagnosed with SLE according to Fries and Holman [33] that met the following criteria: fulfilment of the 2019 EULAR/American College of Rheumatology (ACR) classification criteria for SLE [34], age ≥18 years, no linguistic barriers and residence in the Region of Southern Denmark. We excluded participants without their native lungs. Participants were seen between 1 August 2023 and 31 October 2024.

### Clinical evaluation

Prior to the visit, the participants’ medical records were reviewed focusing on specific patient characteristics such as disease duration, prior treatment, SLE manifestations and signs of pulmonary diseases. At the visit, disease activity was evaluated according to the Systemic Lupus Erythematosus Disease Activity Index 2000 (SLEDAI-2K) [35] and Physicians Global Assessment of Activity (PGA) [36]. Fulfilment of Lupus Low Diseases Activity State (LLDAS) [37] and Definition of Remission in Systemic Lupus Erythematosus (DORIS) [38] were registered. SLE-related damage accrual was registered according to Systemic Lupus Erythematosus International Collaborating Clinics/American College of Rheumatology Damage Index (SDI) [39].

### Pulmonary function tests

PFTs were performed according to current international guidelines [40–42]. Measurements included forced vital capacity (FVC), diffusion capacity for the lung for carbon monoxide (DLCO), 6-min walk distance (6MWD) and oxygen saturation.

### Chest high-resolution computed tomography scan

Participants underwent HRCT on their visit day on a Revolution CT; General Electric Company; Boston, Massachusetts, USA scanner. Acquisition parameters of the diagnostic end-inspiratory scanning are collimation 8 cm, kV 120, SmartmA (140–900 mA), Noise Index 25, Pitch 0.5, Rotation time 0.35 s, Asir-V 40%. Images were reconstructed using a 1024 × 1024 matrix and chest algorithm. Axial slice thickness was 0.625 mm. Image overlap 20%. Nearly the same acquisition parameters were used for the low-dose end-expiratory scanning, but noise index was raised to 30. End-expiratory images were reconstructed according to a standard algorithm, and a single axial series was reconstructed. Slice thickness was 2.5 mm. Examinations were assessed on Vue PACS; Koninklijke Philips N.V., Amsterdam, The Netherlands. We used prior scans, if participants had an HRCT or a chest computed tomography scan (CT) performed within 6 months prior to their study date, and if an experienced radiologist (S.M.W.H.) evaluated that the scan was of sufficient quality. All scans were assessed by S.M.W.H.

### Diagnosing interstitial lung diseases

Based on the above-mentioned investigations, a consensus diagnosis was reached at an MDD regarding the presence and subtype of ILD in accordance with recommendations for ILD [16, 17]. The MDD involved two experienced pulmonologists (J.R.D. and S.M.L.), an experienced thoracic radiologist (S.M.W.H.), and an experienced rheumatologist specialized in SLE (A.V.) and were based on clinical information including HRCT and PFT but blinded to TUS related information.

### Thoracic ultrasound

H.Z.L. performed all TUS scans with a Hitachi Arietta v70 machine, software version 3.0.2., using a convex transducer (C251) 1–5 MHz. Participants were seated upright and scanned according to a 14-zone protocol [43] saving 8 s clips for each zone. The US machine was programmed with pulmonary settings: Depth 10 cm, focus 2 cm, compound settings and harmonics were deactivated, each clip was optimized as common practice. Each video clip was scored by two reviewers (B.K.S. and H.Z.L.), blinded to each other’s scoring and blinded to all other information. Scores included the number of B-lines in every zone and the total number of zones with irregular visceral pleura and/or thickened parietal pleura (Fig. 1, an example of B-lines and irregular visceral pleura). Inconsistencies between the two reviewers were settled by a discussion and if necessary, by a third reviewer (J.R.D.) according to the predefined SAP. Both reviewers are skilled at a European Federation of Societies for Ultrasound in Medicine and Biology (EFSUMB) level 1 [44]. B-lines were defined as vertical artifacts extending from the pleura to the bottom of the image, moving synchronously with pleural movement; irregular visceral pleura was defined as discontinuities in the smooth hyperechoic pleural line; thickened parietal pleura was defined as a hypoechoic line superficial to the visceral pleura unaffected by respiration. According to the SAP, one zone could maximally have 8 B-lines per lung intercostal space (LIS). Confluent B-lines covering the entire LIS, were scored as 8 B-lines. If the total amount of B-lines was ≥10 B-lines, the TUS scan was considered abnormal according to predefined SAP, inspired by prior studies [21, 28].

**Figure 1:**
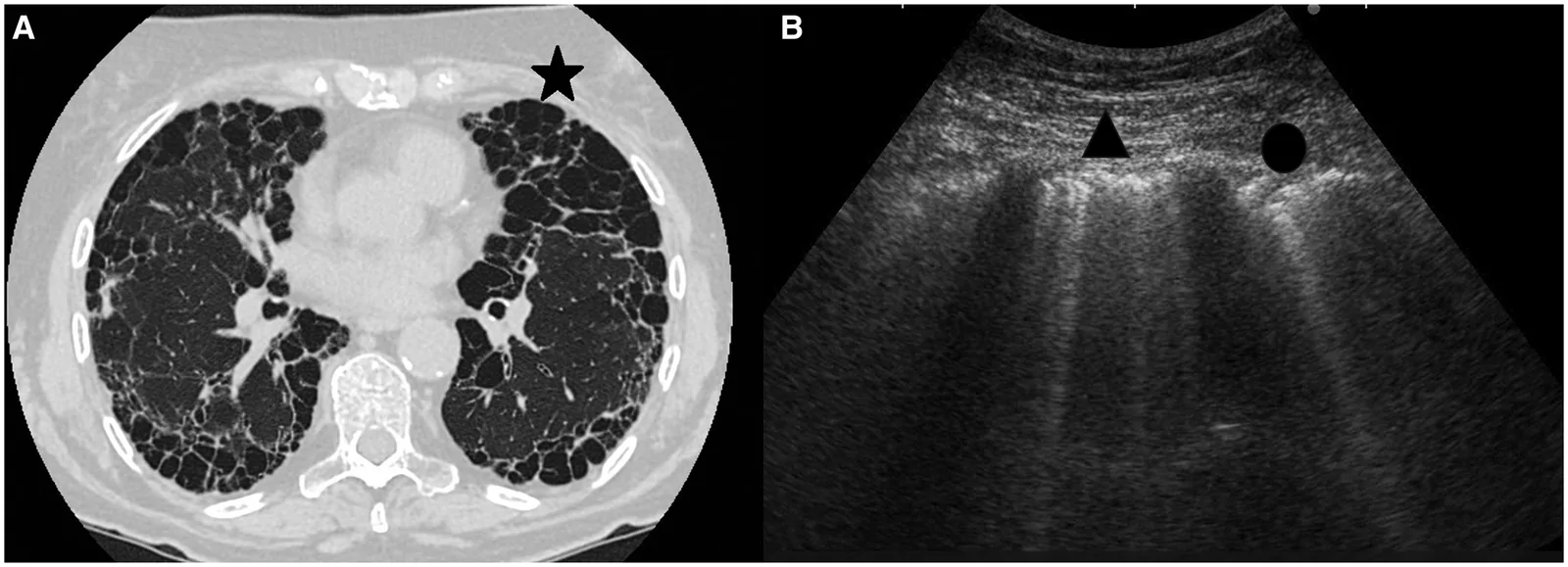
Example of HRCT and corresponding TUS in one patient with fibrotic ILD and SLE. (A) Chest HRCT demonstrating fibrotic ILD in an usual interstitial pneumonia pattern. (B) A corresponding TUS clip demonstrating multiple B-lines and irregular visceral pleura. *, where the TUS is performed; ▲, B-lines originating from pleura; ●, irregular visceral pleura

### Data analysis

We developed and signed an SAP and uploaded it to Zenodo.org (SAP and its deviations are presented in Supplementary Data S2, available at *Rheumatology Advances in Practice* online), prior to the unblinding of data. The analyses were conducted on the full data set, and missing data were handled according to the SAP. Continuous data were described with means and SDs if normally distributed, and medians and interquartile range (IQR) if not. Categorical data were described with frequencies and percentages. Contrast between groups stratified by the presence of ILD was described with standardized difference (Std. Diff), values <0.1 indicate negligible differences and values >0.2 indicate possible meaningful imbalances. In exploratory analyses, we used receiver operating characteristics (ROC) curves and area under the curve (AUC) to explore the diagnostic performance. The optimal threshold for tests was defined by Youden index based on sensitivity and specificity. All diagnostic odds ratios (DOR) were calculated with logistic regression. If one field in the 2 x 2 contingency table included 0, we used the Haldane–Anscombe correction. In logistic regression analyses, we investigated if the number of abnormalities increased risk of ILD. All 95% CI are two-sided and made with log transformations, and data were subsequently exponentiated before reporting. Throughout the manuscript, the 95% CIs are reported in brackets after the estimate.

As TUS might perform poorer among obese individuals, we planned to perform sensitivity analyses excluding participants with body mass indices (BMI) ≥30 kg/m^2^ [28], according to SAP. In addition, we performed several sensitivity analyses. We calculated the positive likelihood ratio (LLR^+^) and negative likelihood ratio (LLR^−^) related to our DTA results. All participants had registrations of primary and secondary outcome measures, why we preformed no analyses of missing values according to SAP. Analyses were performed in Stata BE 18.0 (64 bit).

## Results

### Study population

We identified 318 patients with SLE, 185 (58%) participated (Fig. 2) and 22 were diagnosed with ILD (Supplementary Data S3, available at *Rheumatology Advances in Practice* online). Participants had a mean age of 52 years (SD ±14 years) and were predominantly female 160 (87%). All participants fulfilled SLE classification criteria and most had low disease activity as measured by SLEDAI-2K and accrued damage as registered by the SDI. Participants with ILD had more SDI damage, were older and had lower PFT measures than those without ILD (Table 1). Five participants had a recent chest CT scan of sufficient quality and did therefore not undergo per-protocol scans.

**Figure 2:**
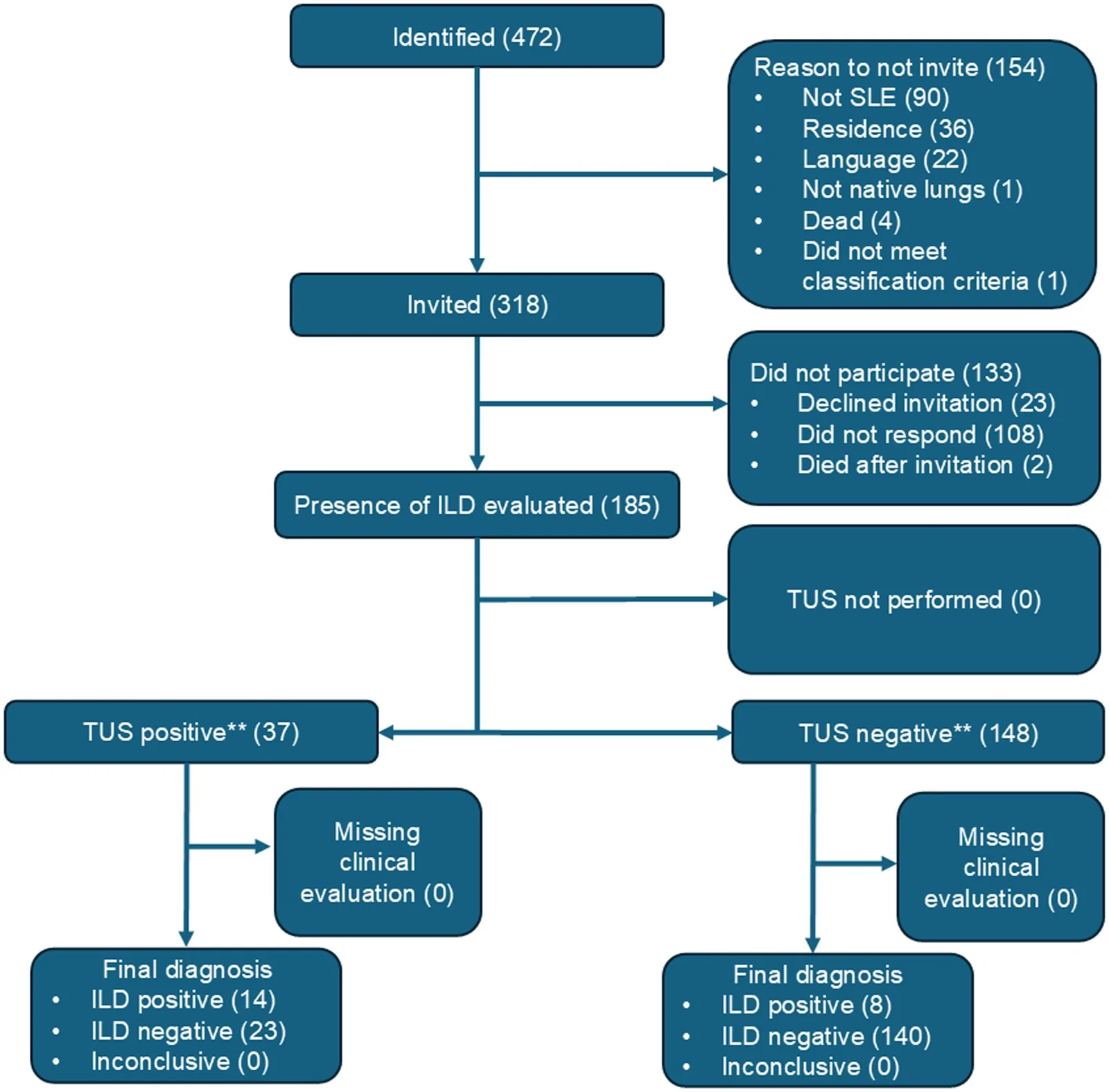
Flowchart illustrating the identification, inclusion, participation, investigation and diagnosis of ILD in the study participants. *See Supplementary material C Appendix S1, for a list of the underlying diseases, available at *Rheumatology Advances in Practice* online. **TUS-positive according to predefined threshold ≥10 B-lines. ILD, interstitial lung diseases; TUS, thoracic ultrasound

**Table 1:** Baseline characteristics for all participants stratified according to presence and absence of ILD.

Baseline characteristics	N	All N = 185	N	ILD Present N = 22	N	ILD Absent N = 163	Std Diff.
Age, years	185	52.0 ± 14.3	22	62.0 ± 16.3	163	50.6 ± 13.5	0.762
Disease duration, years*	185	15.0 (6.5–24.5)	22	21.3 (14.0–30.7)	163	14.6 (5.9–23.9)	0.366
Female sex, no. (%)	185	160 (87%)	22	19 (86%)	163	141 (87%)	0.004
White population, no. (%)	185	173 (94%)	22	21 (96%)	163	152 (93%)	0.096
Smoking, status:	185		22		163		0.235°
Never, no. (%)		86 (47%)		11 (50%)		75 (46%)	0.080
Prior, no. (%)		80 (43%)		11 (50%)		69 (42%)	0.154
Current, no. (%)		19 (10%)		0		19 (12%)	0.514
BMI, kg/m^2^*	184	25.5 (23.0; 28.9)	22	26.9 (23.7; 28.7)	162	25.4 (23.0; 29.0)	0.151
Obese individuals (BMI ≥ 30 kg/m^2^), no. (%)	184	37 (20%)	22	3 (14%)	162	34 (21%)	0.195
*Treatment:*							
Corticosteroid ever, no. (%)	185	167 (90%)	22	22 (100%)	163	145 (89%)	0.498
Hydroxychloroquine ever, no. (%)	185	185 (100%)	22	22 (100%)	163	163 (100%)	0.000
Other csDMARD ever, no. (%)	185	154 (83%)	22	17 (77%)	163	137 (84%)	0.172
bDMARD ever, no. (%)	185	36 (20%)	22	4 (18%)	163	32 (20%)	0.037
*Clinical findings:*							
EULAR/ACR 2019, score 0–51	185	27.1 ± 7.6	22	27.1 ± 7.3	163	27.0 ± 9.7	0.012
SLICC 2012, score 0–17*	185	8 (7; 10)	22	8 (7; 9)	163	8 (7; 10)	0.043
SLEDAI 2K, score 0–105*	185	4 (2; 6)	22	2 (0; 4)	163	4 (2; 6)	0.458
LLDAS achieved no. (%)	185	124 (67%)	22	16 (73%)	163	108 (66%)	0.141
DORIS remission achieved no. (%)	185	69 (37%)	22	8 (36%)	163	61 (37%)	0.022
SDI, score 0–48*	185	1 (1; 3)	22	4 (1; 7)	163	1 (0; 3)	0.896
*Pulmonary functions tests:*							
FVC % of expected*	184	100 (87; 110)	22	89 (71; 99)	162	101 (90; 111)	0.844
FVC low^†^, no. (%)	184	31 (17%)	22	7 (32%)	162	24 (15%)	0.410
DLCO, % of expected	183	74.5 ± 15.5	22	62.4 ± 18.2	161	76.2 ± 13.3	0.866
DLCO low^†^, no. (%)	183	113 (62%)	22	17 (77%)	161	96 (60%)	0.387
*Six min walk test*:							
Distance, % of expected	178	94.1 ± 16.0	20	90.5 ± 18.5	158	94.6 ± 15.6	0.240
Distance low^†^, no. %	178	32 (18%)	20	5 (25%)	158	27 (17%)	0.195
Desaturation below 93%^††^, no. %	146	21 (14%)	19	7 (37%)	127	14 (11%)	0.635
*Thoracic ultrasound:*							
TUS-positive no. (%)	185	37 (20%)	22	14 (64%)	163	23 (14%)	1.180
Number of B-lines, no. 0-112*	185	3 (1; 7)	22	14 (4; 24)	163	3 (1; 6)	0.965
Zones with irregular visceral pleura, no. 0-14*	185	0 (0; 1)	22	2 (0; 3)	163	0 (0; 1)	0.726
Zones with thickened parietal pleura, no. 0-14*	185	0 (0; 1)	22	1 (0; 2)	163	0 (0; 1)	0.595

Values are means ± SDs or median (interquartile range) unless otherwise indicated; Std Diff. refers to the standardized (unitless) difference between ILD present and ILD absent;

* , data is not normally distributed; °, *P*-value calculated with Fischer’s exact test;

† , less than 80% of expected;

†† , below 88% if chronic obstructive pulmonary disease. TUS-positive defined as ≥10 B-lines. N, number; ILD Present, interstitial lung diseases present; ILD Absent, interstitial lung diseases absent; Std Diff., standardized difference; BMI, body mass index; other csDMARD, conventional disease-modifying antirheumatic drugs, not hydroxychloroquine; bDMARD, biologic disease-modifying antirheumatic drugs; EULAR/ACR 2019, European League Against Rheumatism/American College of Rheumatology 2019 classification criteria for SLE [34]; SLICC 2012, Systemic Lupus Erythematosus International Collaborating Clinics classification criteria for SLE [45]; SLEDAI 2K, systemic lupus erythematosus disease activity index 2000 [35]; LLDAS, lupus low disease activity state [37]; DORIS, definition of remission in SLE [38]; SDI, Systemic Lupus Erythematosus International Collaborating Clinics/American College of Rheumatology damage index for SLE [39]; FVC, forced vital capacity; DLCO, diffusing capacity of the lung for carbon monoxide.

### Diagnostic test accuracy analysis of the pre-defined threshold’s association with ILD

At the predefined threshold of ≥10 B-lines, the DOR was 10.7 (3.7–32.3), and at this level the sensitivity was 64% (41–83%), specificity 86% (80–91%), positive predictive value (PPV) 38% (23–55%) and negative predictive value (NPV) 97% (90–98%), Table 2. All 2 x 2 contingency tables are presented in Supplementary Data S4, available at *Rheumatology Advances in Practice* online. An example of TUS abnormalities and corresponding HRCT is shown in Fig. 1.

**Table 2:** DTA outcome measures for TUS using the predefined threshold of ≥10 B-lines for detecting ILD in 185 patients with SLE.

Outcome measure	Results
Diagnostic odds ratio (95% CI)	10.7 (3.7–32.3)
Sensitivity (95% CI)	64% (41–83%)
Specificity (95% CI)	86% (80–91%)
Positive predictive value (95% CI)	38% (23–55%)
Negative predictive value (95% CI)	97% (90–98%)
Positive likelihood ratio (95% CI)	4.5 (2.8–7.4)
Negative likelihood ratio (95% CI)	0.42 (0.24–0.74)
Youden index	0.50

### Exploratory analyses of B-lines’ associations with ILD

According to Youden index, the optimal threshold for the number of B-lines was ≥10, consistent with the predefined threshold. If the threshold for B-lines was increased, the DOR, specificity and PPV increased while sensitivity and NPV decreased, and vice versa (Supplementary Data S5, available at *Rheumatology Advances in Practice* online). The ROC curve differentiated from the line of no-discrimination, and the AUC was 0.81 (0.70–0.91) (Fig. 3). According to logistic regression, the presence of more B-lines made underlying ILD more likely (Supplementary Data S6, available at *Rheumatology Advances in Practice* online).

**Figure 3:**
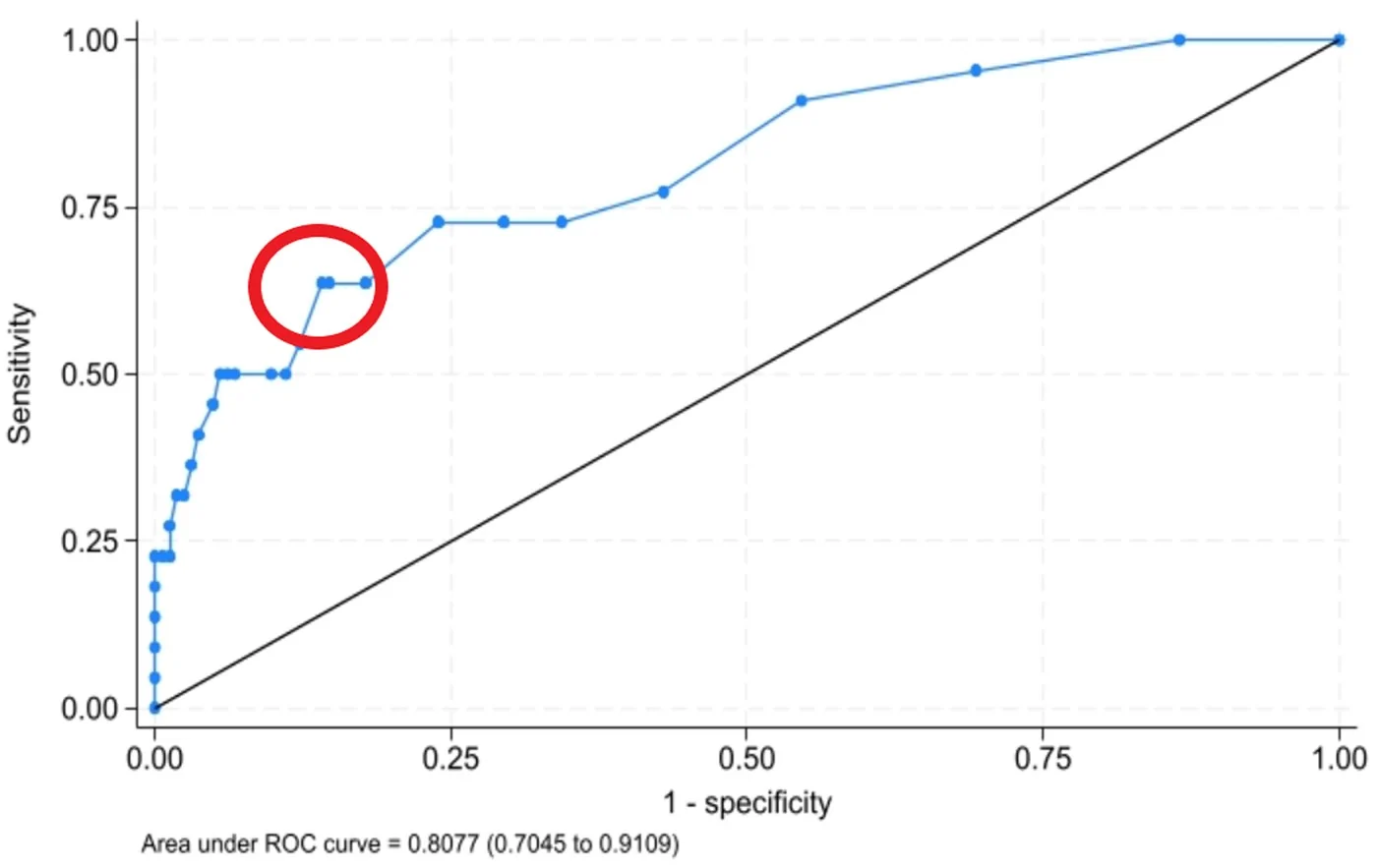
ROC curve and corresponding AUC regarding the B-lines’ association with ILD in 185 patients with SLE. ROC, receiver operating characteristics. Red circle marks the optimal threshold according to Youden index

### Exploratory analysis of association between TUS findings of irregular visceral pleura and/or thickened parietal pleura and ILD

For TUS pleural findings indicative of ILD, we identified an optimal threshold of ≥2 zones with irregular visceral pleura, which was significantly associated with presence of ILD and corresponded to a DOR 4.3 (1.5–12) (Table 3). The presence of more zones with irregular visceral pleura, made underlying ILD more likely (Supplementary Data S7, available at *Rheumatology Advances in Practice* online). Identification of thickening of the parietal pleura by TUS was significantly associated with ILD (Supplementary Data S7, available at *Rheumatology Advances in Practice* online). However, according to AUC, the best-performing TUS modality for identifying ILD was B-lines (Supplementary Data S7, available at *Rheumatology Advances in Practice* online).

**Table 3:** Outcome measures for TUS using abnormal pleura for detecting ILD in 185 patients with SLE.

Association/Outcome measures	≥2 zones with irregular visceral pleura	≥1 zone with thickened parietal pleura
Diagnostic odds ratio (95% CI)	4.3 (1.5–12)	3.7 (1.0–11.9)
Sensitivity (95% CI)	46% (24–68%)	29% (9–48%)
Specificity (95% CI)	81% (74–87%)	90% (86–95%)
Positive predictive value (95% CI)	24% (12–40%)	27% (9–46%)
Negative predictive value (95% CI)	92% (86–96%)	91% (86–95%)
Youden index	0.27	0.19

IVP, irregular visceral pleura; ILD, interstitial lung diseases.

### Sensitivity analyses

Eight out of 22 participants with ILD were not identified with a threshold ≥10 B-lines. Among these, 4 participants had either cystic lung disease or OP, 2 had BMI ≥30 kg/m^2^, and 1 patient had several zones with irregular visceral pleura (Supplementary Data S8, available at *Rheumatology Advances in Practice* online). Among the 23 participants with abnormal TUS but no signs of ILD all but two had other pulmonary diseases including 2 cases of obliterative bronchitis, 8 cases of pleuroparenchymal fibrosis and 11 cases of bronchiectasis (Supplementary Data S9, available at *Rheumatology Advances in Practice* online). Ten out of the 23 also had cardiovascular diseases (Supplementary Data S9, available at *Rheumatology Advances in Practice* online).

We explored the ability of B-lines to identify ILD when excluding participants with characteristics potentially affecting the TUS by means of excluding patients with cystic lung disease, OP and sarcoidosis. Using the threshold of ≥10 B-lines DOR increased from 10.7 to 19.8. Exclusion of participants with a BMI ≥30 kg/m^2^ only slightly increased the DOR to 12.5 (3.79–44.4) (Supplementary Data S9, available at *Rheumatology Advances in Practice* online). Exclusion of both groups of participants (certain types of ILD (DCLD, OP and sarcoidosis) and/or BMI ≥30 kg/m^2^) increased DOR to 34.7, with corresponding PPV 39%, NPV 98%, sensitivity to 86% and specificity to 85% (Supplementary Data S10, available at *Rheumatology Advances in Practice* online). When combining the threshold for B-lines (≥10 B-lines) and zones with irregular visceral pleura (≥1 zone), the sensitivity increased to 73% (50–89%), but the specificity decreased to 56% (48–64%) and DOR decreased to 3.37 (1.2–11.0) (Supplementary Data S9, available at *Rheumatology Advances in Practice* online).

## Discussion

In the present study, the diagnostic accuracy of TUS was evaluated in 185 patients with SLE using a predefined TUS protocol assessing 14 zones on the thorax. The presence of ≥10 B-lines was significantly associated with ILD with a modest test accuracy with DOR 10.7, PPV 38%, NPV 97%, sensitivity 64% and specificity 86%. Thus, a robust association was observed; however, TUS, as implemented using the current protocol, appears to be inadequate to solely detect ILD in patients with SLE.

TUS is seemingly a promising tool to detect findings indicative of underlying ILD in other SARD like rheumatoid arthritis, systemic sclerosis and Sjogrens’ disease with sensitivities ranging from 70% to 98% and specificities ranging from 15% to 98% [20–24, 28]. This is supported by OMERACT in 2024 acknowledging that B-lines and pleural abnormalities are important TUS findings when identifying ILD [46]. Still, the ERS/EULAR state that TUS should not be used to screen for ILD based on literature published before 2022 [12]. In the context of SLE a recently published a case series of recently diagnosed patients with SLE found that the presence B-lines was associated to pulmonary diseases including 3 cases with ILD [27]. In the present DTA study, we showed that ≥10 B-lines were significantly associated with ILD, and performs better than other modalities such as CXR [15] and PFT [9]. However, the DTA performance was modest and cases of ILD were overlooked. To explore characteristics of TUS-positive participants with no ILD and TUS-negative participants with ILD, we performed exploratory analyses.

Our exploratory analyses indicated that certain sub-types of ILD that may occur in SLE were not detected with TUS (cystic lung disease and OP), necessitating the use of alternative ILD screening modalities when these subtypes are suspected. Consistent with this, previous studies have shown that cystic lung disease is not associated with the presence of B-lines [31]. Regarding OP, to our knowledge, no studies have tested the performance of TUS focusing on B-lines. It is possible that OP may not be visualized on TUS by B-lines, since air-filled pulmonary parenchyma often is situated between the OP lesions and the pleura, and furthermore, OP often is localized in segments and not diffusely in the lungs [47]. In agreement with previous studies of TUS, we confirm that TUS focusing on B-lines is less optimal in participants with BMI ≥30 kg/m^2^, although our findings are based on few participants [28].

Excluding participants in whom TUS, as demonstrated in our exploratory analyses, has known limitations, improved the diagnostic accuracy of TUS. Thus, following the exclusion of participants with DCLD, OP and BMI ≥30, the DOR increased to 34.7 and only 2 cases of ILD were missed, hereof 1 with pleural abnormalities and 1 with limited ILD. UIP and NSIP are reported to be the types of ILD, that are associated with the highest mortality in SARD [48], and 9/11 cases were identified using TUS with our predefined B-line threshold alone, among participants with <30 BMI, among the last two one had pleural irregularities and one had limited disease. Therefore, in our results, there is only little evidence to suggest that TUS has reduced DTA in patients with severe ILD. Considerations on this issue should be addressed in future studies.

Though 23 out of 37 TUS-positive patients with SLE did not have ILD, all but two positive had clinically relevant pulmonary disease, indicating that the majority of the TUS-positive patients with SLE could benefit of further diagnostic workup for pulmonary diseases. In theory, TUS should not be able to identify airway diseases [43], but ≥10 B-lines have been reported among patients with airway diseases in another study [28]. We hypothesize, that some airway diseases might affect the parenchymal adjacent to the pleura such as emphysema and obliterative bronchiolitis. Ten on the 23 TUS-positive participants that did not have ILD had cardiovascular disease, but only few had heart failure.

Irregular visceral pleura has been associated with ILD in other SARD [22, 25, 28, 49], but to our knowledge not in relation to SLE. In the present study, ≥2 zones demonstrating irregular visceral pleura were significantly associated with ILD, and an increasing number of zones with irregular visceral pleura increased the likelihood of underlying ILD. As pleuritis is a frequent and severe manifestation of SLE, it is interesting that it has been suggested that pathologies of the parietal pleura can be visualized with TUS among patients with SARD [43]. In the present study, we found an association between thickened parietal pleura and ILD in SLE. Our findings indicate that ILD might be considered in SLE patients with TUS demonstrating thickening of the parietal pleura, but our results are based on our exploratory analyses and require to be reproduced. In future studies, it should be explored in more detail if pleural disease and ILD in SLE share a common pathogenesis, as prior pleuritis has been associated with presence of SLE-ILD [9].

Like TUS, measurement of circulating biomarkers in blood samples is an easily accessible method to identify SLE patients at risk of ILD in an outpatient clinic setting. In a recent systematic review, an association between pulmonary diseases and some biomarkers in SLE was found [50]. The most robust association was between anti-double-stranded DNA antibodies and pulmonary diseases, DOR 1.84 (1.16–2.92), but no significant associations between biomarkers and ILD were found.

Currently, the international recommendations for diagnosing ILD in SARD is a MDD based on several factors including PFT and HRCT. Our study underscores that TUS at present cannot replace an MDD, but TUS may be an easy-accessible tool to identify patients who need a further diagnostic workup due to indicative ILD, as TUS seemingly perform better than CXR, biomarkers and PFT [9, 15, 50].

The strengths of this study were that participants were unselected out-patients, making results most relevant to out-patient clinics and limiting the selection bias. The reference standard, HRCT followed by MDD diagnosis of ILD, was according to recommendations [16]. TUS was performed using a standardized protocol and results were evaluated by two independent and experienced ultrasound sonographers blinded to other information which limits the risk of bias. However, the study was performed in a single centre on a limited number of participants including a limited number of ILD cases. Five participants did not undergo per-protocol HRCT on the study day as recent scans were present, but the scans were evaluated to be of good quality, why we do not believe it affects the overall result. Some findings were based on exploratory analyses and must be interpreted with caution until other studies confirm the results.

### Patients’ perspective

It is important that TUS is good at excluding ILD based on B-lines. It is comforting and important for patients that the limitations of the method are mentioned explicitly. The patient partner is wondering how the findings can be implemented. Maybe TUS could be used for screening? Or in symptomatic patients? The patient partner experienced the TUS as easy and comfortable compared with other investigations such as HRCT and PFT.

We asked the participants how they experienced TUS. 182 out of 183 would undergo TUS again if a doctor recommended it. We asked 182 participants about how bothersome the TUS were on a scale from 0 to 10 patients (0—‘not bothering at all’, 10—‘worst experience ever’), mean of 0.40 SD 0.88.

## Conclusion

In SLE, TUS focusing on presence of ≥10 B-lines demonstrated modest DTA and high NPV for ILD. Our exploratory analyses indicate that certain types of SLE-related ILD (cystic lung diseases and OP) and BMI ≥30 kg/m^2^ adversely affect the DTA of TUS. In exploratory analyses, both irregular visceral pleura and thickening of the parietal pleura were significantly associated with ILD warranting further studies.

## Supplementary Material

rkag126_Supplementary_Data

## Data Availability

The data underlying this article will be shared on reasonable request to the corresponding author.
