# Supplementary material for: Thoracic ultrasound finding interstitial lung disease in SLE: a diagnostic test accuracy study
Source: Rheumatol Adv Pract. 2026 Oct 14;10(4):rkag126. doi: 10.1093/rap/rkag126 (PMC13713183; doi:10.1093/rap/rkag126)
Supplement: Supplementary file 1 [file rkag126_supplementary_data.zip › Supplementary Data S2 SAP and deviations from SAP.pdf]

Deviation from SAP

In general, we removed pleural diseases, pulmonary diseases and airway diseases throughout the manuscript, to decrease the amount of data, and make the paper more readable and easier to comprehend. Results of pleural diseases, pulmonary diseases and airway diseases will be presented in another paper.

Objectives.

In 2) and 3) pleural diseases pulmonary diseases and airway diseases were removed.

DOR was changed to “diagnostic test utility”, “explore the utility” to make it fit better with the diagnostic accuracy test format. In diagnostic utility DOR is included, why we still report the proposed outcome.

Presenting results.

We included positive and negative likelihood ratio to the diagnostic accuracy test results, as we found it a relevant measure.

As we narrowed in the manuscript to only focus on ILD, we changes the flow-chart – figure 1, to only report ILD.

The suggested mock-up table 2 is presented in collected in supplementary materials. We did this to reduce the amount of data presented in the main text to improve the readability of the manuscript.

To the suggested mock-up figure 3 and table 3 are removed, as we removed all concerning pleural diseases, pulmonary and airway diseases.

The proposed mock-up figure 3 has been moved to appendix 3, because Rheumatology only allows 6 tables or figures in the manuscript, and we found that this was the most fitting in an appendix.

We have included a figure 2, to illustrate how thoracic ultrasound abnormalities look, this was done to help readers not familiar with thoracic ultrasound to better understand the manuscript.

We present table 3 with a summary of diagnostic characteristics of outcome measures for TUS using exploratory thresholds for B-lines and zones with irregular visceral pleura for detecting ILD in 185 patients with SLE. This was to present the data in an easily readable format.

Supplementary materials, appendices in the SAP have been renamed and are now called supplementary materials. We present the proposed appendices, and several newly developed, where we explore the test characteristics of b-lines to identify TUS.

We did not perform any best case / worst case scenarios as our primary and secondary results were measured, why we did not make the proposed mock-up appendix 2a and 2b.

Statistical Analysis Plan

Thoracic Ultrasound for Detecting Interstitial Lung Diseases and other Pulmonary Diseases in patients with Systemic Lupus Erythematosus: An Exploratory Diagnostic Test Accuracy Study

Administrative

Clinical trials number: S-20210164

Ethics committee number: 23/22079

Danish data protection agency number: NCT06087523

Version: 01

Date: 05.08.2025

Roles

Statistical analyst and writer of statistical analysis plan: Henrik Zachar Langkilde, MD

Statistical advisor: Robin Christensen; BSc, MSc, PhD; Professor of Biostatistics & Clinical Epidemiology

Principle investigator: Anne Voss, MD, PhD; Assoc. Professor of Rheumatology

Collaborators

Bjørk Sofiudottir, MD, PhD

Stefan Harders, MD, PhD

Jesper Rømhild Davidsen, MD, PhD

Stefan Møller Luef, MD

Sille Fløjborg, AP

Signatures

Henrik Zachar Langkilde (Principal investigator)

Date and signature: 5/8-2025

Robin Christensen (Senior biostatistician)

Date and signature: 2025, Aug 05

Anne Voss (Sponsor)

Date and signature: 5/8 2025

Purpose of the SAP

To describe the statistical analysis plan (SAP) for Thoracic Ultrasound for Detecting Interstitial Lung Diseases and other Pulmonary Diseases in Systemic Lupus Erythematosus study. This SAP was prespecified and developed to secure transparency and reveal how we intend to perform and report the statistical analyses a priori focusing on the main parts of the manuscript.

INTRODUCTION

Systemic Lupus Erythematosus (SLE) is an autoimmune disease, that can affect every organ system (1). Pulmonary diseases (PD) are common in SLE and covers several conditions including interstitial lung diseases (ILD), pleural diseases, and airway diseases (2). Diagnosing PD in SLE can be cumbersome as it often requires specialized tests and personal (3) and difficult because symptoms range from severe to vague or not present at all (2). Early diagnosis of PD in SLE is important as studies indicates that treatment improves outcomes (4, 5). Thoracic ultrasound (TUS) shows promise as a tool to identify PD (especially ILD) among patients with other rheumatic disease (6-8), but have only been sparsely investigated in SLE (6). The studies have primarily focused on the presence of B-lines, defined as a sharp hyperechoic line, that originates from the pleura and stretch across the whole picture, moving synchronously with the movement of the lung.

In diagnostic test accuracy studies, the reference standard (gold standard) is the best available method for determining true disease status. It serves as the benchmark for evaluating the performance of the index test (test under investigation). An ideal reference standard is highly accurate, minimizing false positives and false negatives, and widely accepted, meaning it is recognized as the best diagnostic method for the condition. It should also be independent of the index test to prevent incorporation bias.

In this study, TUS is the index test, the reference standard is diagnoses of ILD, other PD, such as pleural diseases, and airway diseases from multidisciplinary discussion (MDD) based on High-Resolution Computed Tomography scans of thorax (HRCT), pulmonary function tests, clinical examination, and review om medical record.

Rationale

PD is a frequent and potentially severe manifestation of SLE, yet its diagnosis remains challenging due to the heterogeneity and often subclinical nature of symptoms. Current diagnostic approaches rely on advanced imaging and specialist evaluation, which may limit early detection. Thoracic ultrasound is a non-invasive, accessible, and radiation-free modality that has shown diagnostic value in identifying interstitial lung disease (ILD) and other pulmonary complications in various rheumatic diseases. However, its utility in SLE-associated PD remains insufficiently explored. Establishing the diagnostic accuracy of TUS, particularly the presence of B-lines and but also other sonographic features, could enhance early detection and management of PD in SLE, thereby improving patient outcomes.

Objectives

The primary objective is to investigate the diagnostic Odds Ratio (DOR) of B-lines for identifying ILD in SLE.

The secondary objectives are to investigate:

- The DOR of B-lines for identifying PD, pleural diseases, and airway diseases in SLE
- The DOR of pleural irregularities and pleural thickness for identifying ILD, PD, pleural disease, and airway diseases

METHODS

Study design

The study will be reported according to Standard for Reporting Diagnostic Accuracy (STARD) statement (9). To address our research question, we formulated the following PICO (Population, Index test, Comparison, and Outcome) questions:

- Population. Adult SLE patients
- Index test. TUS
- Comparison (Reference standard). Diagnoses of ILD, PD, pleural diseases, and airway diseases according to MDD
- Outcome. The DOR of the index test for diagnosing reference standard

Data were collected in a predefined Research Electronic Data Capture (REDCap) database and stored blinded until this SAP is signed.

Study design: A diagnostic accuracy and exploratory, prospective, population-based, and cross-sectional study

Eligibility criteria: We used the following inclusion criteria:

- A diagnosis of SLE
- Fulfilment of the EULAR/ACR 2019 classification criteria (10)
- Age ≥ 18 years
- Ability to understand, read, and speak Danish
- Residence in Region Southern Denmark

Patients were not eligible if they did not have native lungs.

Patient recruitment: SLE patients identified by a search in all hospitals in Region Southern Denmark's IT systems, for visits in the period 1. July 2021 to 30. June 2023 coded as related to SLE or SLE related diseases. Study visits between 1. August 2023 and 31. October 2024.

Patient population: We aimed to include all patients with SLE in Region Southern Denmark (with approximately 1,200,000 inhabitants). We estimate how a desired size of study population by a sample size calculation. We expect that the prevalence of PD and the subtypes of PD we investigate range from 10-60%. With a desired confidence level of 95% and a margin of error of 10%.

Maximal prevalence:

$$n = \frac{Z^2 \times P(1-P)}{d^2} = \frac{1.96^2 \times 0.60(1-0.60)}{0.10^2} = \frac{3.8416 \times 0.240}{0.01} = 92.20\ round\ up\ to\ 93$$

Maximal number of participants needed, as 50% is included in the interval:

$$n = \frac{Z^2 \times P(1-P)}{d^2} = \frac{1.96^2 \times 0.50(1-0.50)}{0.10^2} = \frac{3.8416 \times 0.250}{0.01} = 96.04 \textit{ rounded up to } 97$$

Minimal prevalence:

$$n = \frac{Z^2 \times P(1-P)}{d^2} = \frac{1.96^2 \times 0.10(1-0.10)}{0.10^2} = \frac{3.8416 \times 0.0900}{0.01} = 34.57 \textit{ round up to } 35$$

P = estimated prevalence, d = desired precision (margin of error), Z = z-score for your confidence level.

We estimated that we needed at least 97 participants.

Index test: ***Thoracic ultrasound, TUS.*** A 14-zone protocol (11) was used by HL saving eight second video clips from each zone. The video clips were later evaluated for presence of B-lines, irregularities of the visceral pleura, and thickening of the parietal pleura by HL and BS blinded to each other's results and results of the MDD. Disagreements in regards of the scoring of TUS clips were settled in a discussion between HL and BS and when relevant the TUS clips were evaluated by JD for final decision. Discrepancies in the scoring of TUS clips between BS and HZL will be resolved as follow:

BS and HZL will review and discuss individual video clips where discrepancies arise concerning the participant's status in relation to:

- Status TUS positive or negative in regards to our definition regarding B-lines
- Discrepancy exciding 20% in the total count of B-lines*
- Number of zones with irregularities of the visceral pleura
- Number of zones with thickness of the parietal pleura

* A 20% threshold has been pragmatically selected, because we deem differences below this margin as clinically insignificant. For instance, a difference between 13 and 15 B-lines in total in a participant is not considered clinically relevant. The use of percentages ensures proportionality in assessment. In cases where the discrepancy in B-line counts is less than 20%, the average number of B-lines will be utilized.

Reference standard: ***Multidisciplinary discussion, MDD.*** At a MDD the participants were diagnosed with or without PD and PD was subcategorized as having ILD, airway diseases, or pleural diseases. The diagnoses of PD were performed blinded to the scoring of the TUS and based on information from HRCT, pulmonary function tests (including six-minute walk test, spirometry, body plethysmography, and diffusion test), clinical examination, and information from the participants' medical record. At the MDD AV, JD, SL, and SH participated.

Efforts to avoid bias: Blinding. Data regarding HRCT scans and diagnoses of ILD, PD, and other subtypes of PD and TUS are stored in a Research Electronic Data Capture (REDCap) database, and never unblinded at the same time, until this SAP is signed. BS only has access to a dedicated TUS evaluation tool in the REDCap database, and is completely blinded to all clinical information. While HL and JD fill in data on TUS, they have no access to MDD diagnoses in REDCap.

Defining outcomes: ***Outcomes – diagnostic odds ratio regarding the association between different TUS modalities and the presence of interstitial lung diseases (ILD) and other pulmonary diseases (PD).*** The outcomes are the DOR of the index tests' (TUS) ability to diagnose the reference standard (MDD). We seek to explore different TUS modalities' (b-lines, irregular pleura, thickened pleura) ability to identify ILD, PD, pleural diseases, and airway diseases.

Statistical analyses

Analysis of the population

The analyses will be conducted as intention to survey, that is, analyses will be performed on the full data set. Participants will be stratified according to presence or absence of ILD, PD, pleural diseases, and airway diseases. In mock-up Figure 1 we describe how we plan to present the flow of possible study participants.

Baseline characteristics and prevalence of TUS positive

Mock-up Table 1. We will describe selected baseline characteristics of the included study participants. Continues data will be described with means and standard deviations (SD) if normal distributed and medians and interquartile range if not. We plan to investigate if data is normal distributed with Shapiro-Wilks test (defining p-value above 0.05 as a sign of normal distribution) and histograms. Categorical data will be described with frequencies and percentages. Contrast participants stratified according to presence of ILD will be described with Standardised difference. TUS positive is defined as ≥10 B-lines as the primary measure. We will also report number of B-lines and number of zones with irregular visceral pleura and thickened parietal pleura.

Exploring test performances in relation to interstitial lung diseases (ILD)

Mock-up Figure 2 and Table 2. The diagnostic performance for B-lines in diagnosing ILD has only been sparsely investigated in SLE, why we plan to investigate how number of B-lines performs with Receiver Operating Characteristics (ROC) curves regarding identifying ILD. We will investigate the performance of B-lines visually with ROC-curves and additionally with Area Under the Curve (AUC), and the optimal threshold defined by the highest Youden index. We will present DOR with 95% Confidence Intervals (95%CI), sensitivity, specificity, positive predictive value (PPV), negative predictive value (NPV), and Youden index at the optimal threshold and other predefines thresholds.

Exploring test performances in relation to pulmonary diseases and subtypes of pulmonary diseases

Mock-up Figure 3 and Table 3. We plan to investigate the DOR regarding B-lines in diagnosing PD, pleural diseases, and airway diseases in SLE. We plan to do this by ROC curves in mock-up Figure 3 and related numbers in Table 3. In relation to the ROC curves in Figure 3 we plan to present the corresponding AUC and find the optimal threshold defined by Youden index. In Table 3 we will present for the optimal number B-lines and several other predefined number of B-lines for PD, pleural diseases, and airway diseases: DOR with 95%CI, sensitivity, specificity, positive predictive value (PPV), negative predictive value (NPV), and Youden index.

Performance of B-lines, pleural irregularities, and pleural thickness in diagnosing ILD, PD, pleural diseases, and airway diseases will be investigated with univariate logistic regression analyses, and a DOR will be presented as proposed in Appendix 1.

Missing data

Missing index test or reference standard will lead to exclusion from the primary analysis. We expect data to be missing at random. Should missing data occur concerning our primary or secondary outcomes, we plan to examine its potential effect through best-case and worst-case scenario analyses and present the scenarios in an appendix, mock-up Appendix 2a and Appendix 2b.

Sensitivity analysis

A study has shown that TUS performs poorer among obese participants, why we plan to perform sensitivity analysis to investigate the effect of obesity on TUS performance in that particular subgroup of patients. We plan to define obesity as BMI ≥ 30 kg/m^2 inspired by Sofíudóttir et *al.* who found that TUS performed poorer among participants with BMI > 32 (7), mock-up Appendix 3.

Statistical software

We plan to analyse all data with Excel and Stata BE 18.0 (64bit).

RESULTS – Anticipated Manuscript Outline (Main Analyses)

Flow of participants in the study in a flowchart as shown in Figure 1.

Mock-up Figure 1. Proposed flow-chart describing the trial flow of possible participants in the study.

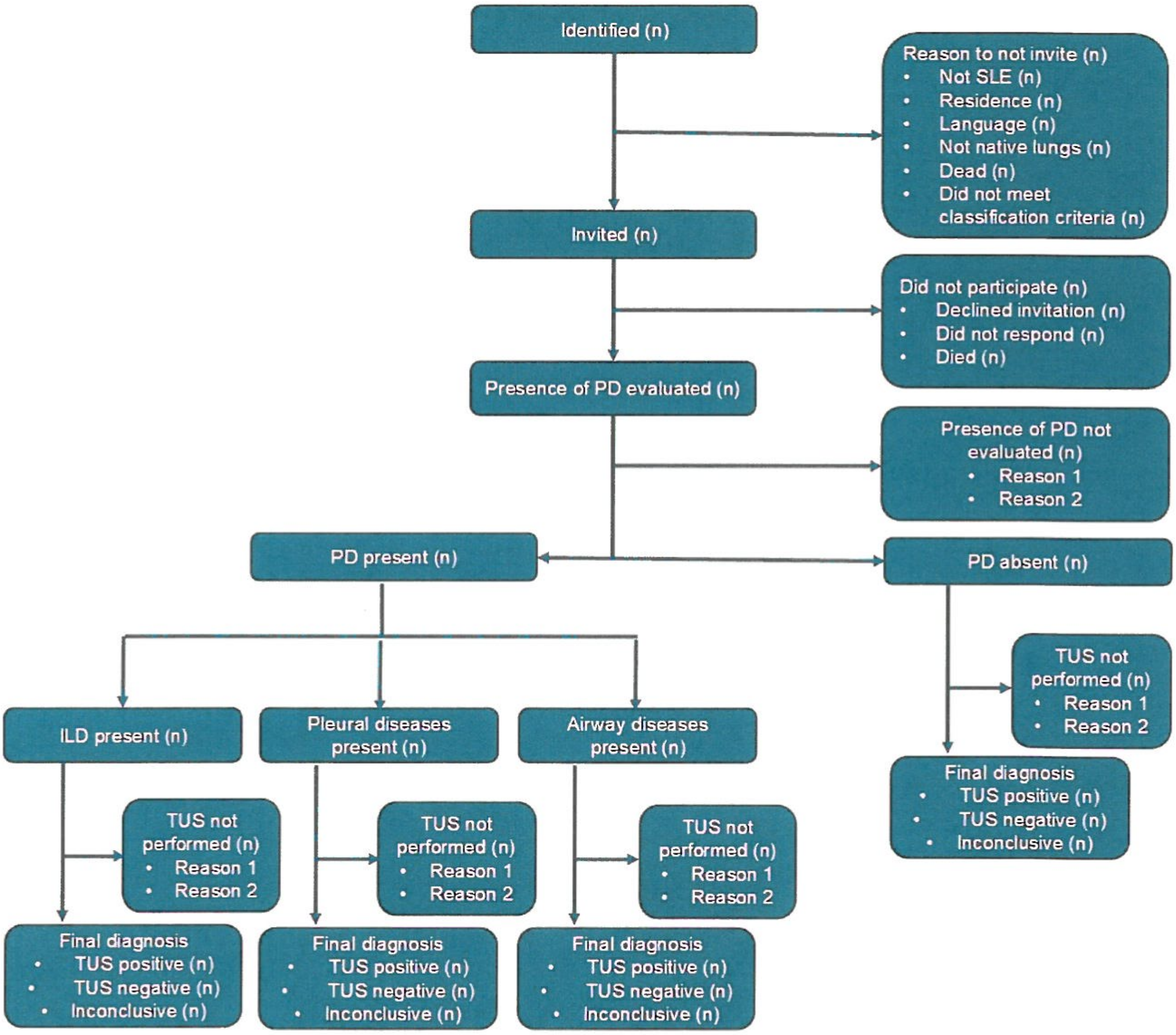

SLE – Systemic lupus erythematosus; PD – Pulmonary diseases; ILD – Interstitial lung diseases; TUS – Thoracic ultrasound (defined by number of b-lines); TUS positive ≥ 10 B-lines.

Mock-up Table 1. Baseline characteristics, for all patients, and stratified according to presence and absence of interstitial lung diseases

**Baseline characteristics**	**No.**	**All**	**ILD Present**	**ILD Absent**	**Std Diff.**
Age, years					
Disease duration, years					
Female sex, no. (%)					
White population, no. (%)					
Smoking, status:<br>Never, no. (%)<br>Prior, no. (%)<br>Present, no. (%)					
BMI, kg/m^2					
Obese individuals (BMI≥30 kg/m^2), no. (%)					
Treatment:					
Corticosteroid ever, no. (%)					
Hydroxychloroquine ever, no. (%)					
Other csDMARDs ever, no. (%)					
BioDMARDs ever, no. (%)					
Clinical findings:					
EULAR/ACR 2019, score 0-51					
SLICC 2012, score 0-17					
SLEDAI 2K, score 0-105					
LLDAS achieved no. (%)					
DORIS remission achieved no. (%)					
SLICC DI, score 0-48					
Body plethysmography and diffusion test					
FVC % of expected					
FVC low*, no. (%)					
DLCO, % of expected					
DLCO low*, no. (%)					
6 min walk test:					
Distance, % of expected					
Distance low*, no. %					
Desaturation below 93%**, no. %					
Thoracic ultrasound					
TUS positive					
Number of B-lines, no. 0-112					
Zones with irregular visceral pleura, no. 0-14					
Zones with thickened parietal pleura, no. 0-14					

Values will be means and SDs unless otherwise indicated; * - less than 80% of expected; ** - below 88% if chronic obstructive pulmonary disease. No. – Number; ILD Present – Interstitial lung diseases present; ILD Absent – No interstitial lung diseases; Std Diff – Standardised Difference; BMI – Body Mass Index; csDMARD – Conventional Disease-Modifying Antirheumatic Drugs; other csDMARD – Conventional Disease-Modifying Antirheumatic Drugs, not hydroxychloroquine; bioDMARDs – Biologic Disease-Modifying Antirheumatic Drugs. EULAR/ACR 2019 – European League against Rheumatism / American College of Rheumatology 2019 Classification Criteria (10); SLICC 2012 - Systemic Lupus International Collaborating Clinics classification criteria for Systemic Lupus Erythematosus (12); SLEDAI 2K – Systemic Lupus Erythematosus Disease Activity Index 2000 (13); LLDAS – Lupus Low

Disease Activity State (14); DORIS – Definition of Remission in SLE (15); SLICC DI – Systemic Lupus International Collaborating Clinics /American College of Rheumatology Damage Index for Systemic Lupus Erythematosus (16); FVC – Forced Vital Capacity; DLCO - Diffusing capacity of the Lung for Carbon Monoxide. TUS positive defined as ≥ 10 B-lines.

Mock-up Figure 2. Proposed Receiver Operating Characteristics Curve of number of b-lines' ability to identify interstitial lung diseases in systemic lupus erythematosus.

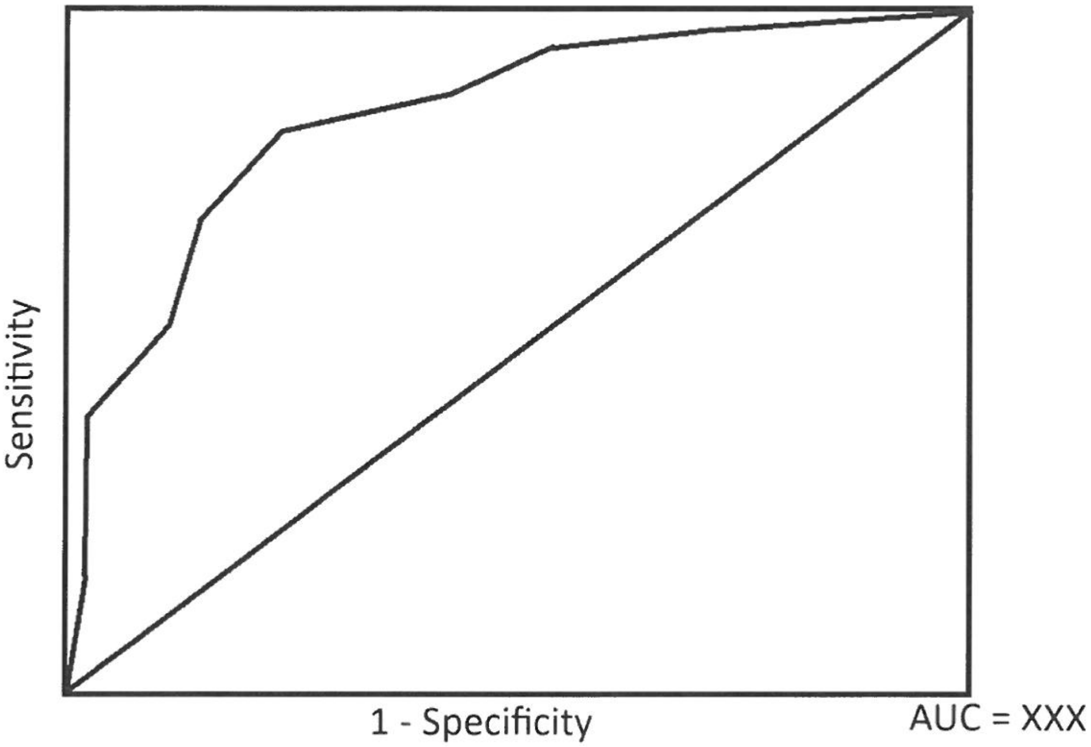

AUC – Area under the curve

Mock-up Table 2. Proposed summary of Receiver Operating Characteristics (ROC) of the associations between B-lines and interstitial lung diseases and the performance of the optimal threshold and other numbers of B-lines in interstitial lung diseases.

Threshold, number of b-lines	DOR, 95% CI	Sensitivity, 95% CI	Specificity, 95% CI	PPV, 95% CI	NPV, 95% CI	Youden Index
Interstitial lung diseases						
Optimal threshold according to ROC curve						
5						
8						
9						
10						
11						
12						
15						
20						
25						

DOR – Diagnostic Odds Ratio; 95%CI – 95% confidence interval, PPV – positive predictive value; NPV – negative predictive value.

Mock-up Figure 3. Proposed receiver operating characteristics curves and corresponding area under the curve for number of B-lines in pulmonary diseases, pleural diseases, and airway diseases in systemic lupus erythematosus.

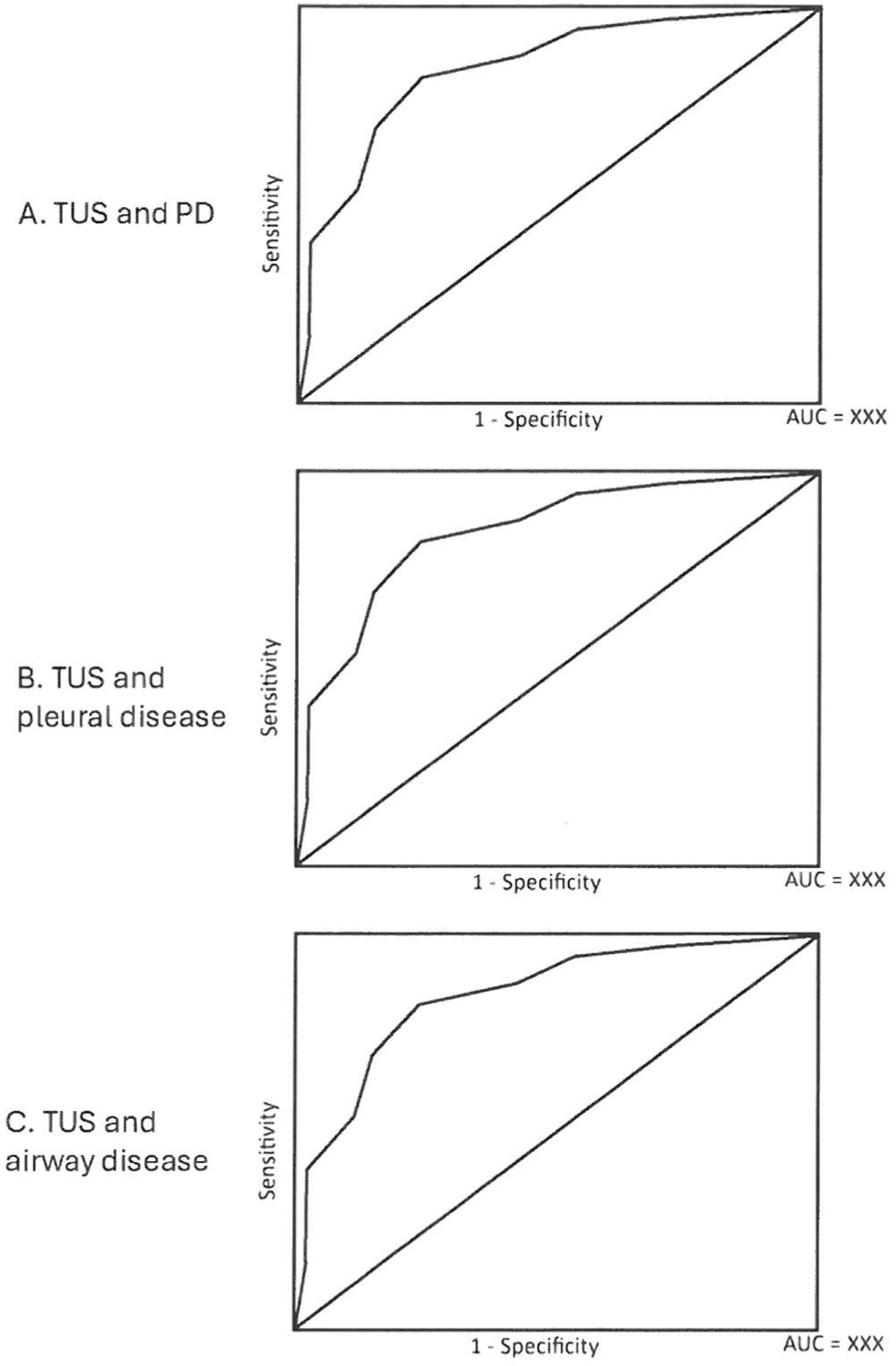

TUS – Thoracic ultrasound; PD – pulmonary diseases; AUC – Area Under the Curve.

Mock-up table 3. Summary of Receiver Operating Characteristics of the diagnostic performance of different numbers of B-lines in diagnosing pulmonary diseases, pleural diseases, and airway diseases in systemic lupus erythematosus.

Threshold, number of b-lines	DOR, 95% CI	Sensitivity, 95% CI	Specificity, 95% CI	PPV, 95% CI	NPV, 95% CI	Youden Index
Pulmonary diseases, with different cutoffs						
Best threshold according to ROC curve						
5						
8						
9						
10						
11						
12						
15						
20						
25						
Airway diseases, with different cutoffs						
Best threshold according to ROC curve						
5						
8						
9						
10						
11						
12						
15						
20						
25						
Pleural diseases, with different cutoffs						
Best threshold according to ROC curve						
5						
8						
9						
10						
11						
12						
15						
20						
25						

DOR – Diagnostic Odds Ratio; 95% CI – 95% confidence interval, PPV – positive predictive value; NPV – negative predictive value.

References

1. Kaul A, Gordon C, Crow MK, Touma Z, Urowitz MB, van Vollenhoven R, et al. Systemic lupus erythematosus. Nat Rev Dis Primers. 2016;2:16039.

2. Bendstrup E, Lynn E, Troldborg A. Systemic Lupus Erythematosus-related Lung Disease. Semin Respir Crit Care Med. 2024.

3. Kondoh Y, Makino S, Ogura T, Suda T, Tomioka H, Amano H, et al. 2020 guide for the diagnosis and treatment of interstitial lung disease associated with connective tissue disease. Respir Investig. 2021;59(6):709-40.

4. Traynor AE, Corbridge TC, Eagan AE, Barr WG, Liu Q, Oyama Y, et al. Prevalence and reversibility of pulmonary dysfunction in refractory systemic lupus: improvement correlates with disease remission following hematopoietic stem cell transplantation. Chest. 2005;127(5):1680-9.

5. Duron L, Cohen-Aubart F, Diot E, Borie R, Abad S, Richez C, et al. Shrinking lung syndrome associated with systemic lupus erythematosus: A multicenter collaborative study of 15 new cases and a review of the 155 cases in the literature focusing on treatment response and long-term outcomes. Autoimmun Rev. 2016;15(10):994-1000.

6. Xie HQ, Zhang WW, Sun S, Chen XM, Yuan SF, Gong ZH, et al. A simplified lung ultrasound for the diagnosis of interstitial lung disease in connective tissue disease: a meta-analysis. Arthritis Res Ther. 2019;21(1):93.

7. Sofíudóttir BK, Harders S, Laursen CB, Lage-Hansen PR, Nielsen SM, Just SA, et al. Detection of Interstitial Lung Disease in Rheumatoid Arthritis by Thoracic Ultrasound: A Diagnostic Test Accuracy Study. Arthritis Care Res (Hoboken). 2024;76(9):1294-302.

8. Vermant M, Kalkanis A, Goos T, Cypers H, De Crem N, Neerinckx B, et al. Ultrasonographic Presentation and Anatomic Distribution of Lung Involvement in Patients with Rheumatoid Arthritis. Diagnostics (Basel). 2023;13(18).

9. Bossuyt PM, Reitsma JB, Bruns DE, Gatsonis CA, Glasziou PP, Irwig L, et al. STARD 2015: an updated list of essential items for reporting diagnostic accuracy studies. Bmj. 2015;351:h5527.

10. Aringer M, Costenbader K, Daikh D, Brinks R, Mosca M, Ramsey-Goldman R, et al. 2019 European League Against Rheumatism/American College of Rheumatology classification criteria for systemic lupus erythematosus. Ann Rheum Dis. 2019;78(9):1151-9.

11. Laursen CB, Sloth E, Lassen AT, Christensen R, Lambrechtsen J, Madsen PH, et al. Point-of-care ultrasonography in patients admitted with respiratory symptoms: a single-blind, randomised controlled trial. Lancet Respir Med. 2014;2(8):638-46.

12. Petri M, Orbai AM, Alarcón GS, Gordon C, Merrill JT, Fortin PR, et al. Derivation and validation of the Systemic Lupus International Collaborating Clinics classification criteria for systemic lupus erythematosus. Arthritis Rheum. 2012;64(8):2677-86.

13. Gladman DD, Ibañez D, Urowitz MB. Systemic lupus erythematosus disease activity index 2000. J Rheumatol. 2002;29(2):288-91.

14. Franklyn K, Lau CS, Navarra SV, Louthrenoo W, Lateef A, Hamijoyo L, et al. Definition and initial validation of a Lupus Low Disease Activity State (LLDAS). Ann Rheum Dis. 2016;75(9):1615-21.

15. van Vollenhoven RF, Bertsias G, Doria A, Isenberg D, Morand E, Petri MA, et al. 2021 DORIS definition of remission in SLE: final recommendations from an international task force. Lupus Sci Med. 2021;8(1).

16. Gladman D, Ginzler E, Goldsmith C, Fortin P, Liang M, Urowitz M, et al. The development and initial validation of the Systemic Lupus International Collaborating Clinics/American College of Rheumatology damage index for systemic lupus erythematosus. Arthritis Rheum. 1996;39(3):363-9.

Appendix 1. Univariate regression analyses comparing diagnostic odds ratio of different thoracic ultrasound measures modalities when identifying interstitial lung diseases, pulmonary diseases, pleural diseases, and airway diseases in systemic lupus erythematosus.

TUS modality	DOR (95% CI)
Interstitial lung diseases	
B-lines, normal/abnormal	
B-lines, continuous	
Irregular visceral pleura, normal/abnormal	
Irregular visceral pleura, continuous	
Thickened parietal pleura, normal/abnormal	
Thickened parietal pleura, continuous	
Pulmonary diseases	
B-lines, normal/abnormal	
B-lines, continuous	
Irregular visceral pleura, normal/abnormal	
Irregular visceral pleura, continuous	
Thickened parietal pleura, normal/abnormal	
Thickened parietal pleura, continuous	
Pleural diseases	
B-lines, normal/abnormal	
B-lines, continuous	
Irregular visceral pleura, normal/abnormal	
Irregular visceral pleura, continuous	
Thickened parietal pleura, normal/abnormal	
Thickened parietal pleura, continuous	
Airway diseases	
B-lines, normal/abnormal	
B-lines, continuous	
Irregular visceral pleura, normal/abnormal	
Irregular visceral pleura, continuous	
Thickened parietal pleura, normal/abnormal	
Thickened parietal pleura, continuous	

TUS - Thoracic ultrasound; DOR – Diagnostic Odds ratio; 95% CI – 95% confidence interval

Mock-up Appendix 2a. Best-case scenario. Univariate regression analyses comparing diagnostic odds ratio of different thoracic ultrasound measures modalities when identifying interstitial lung diseases, pulmonary diseases, pleural diseases, and airway diseases in systemic lupus erythematosus.

TUS modality	DOR (95% CI)
Interstitial lung diseases	
B-lines, normal/abnormal	
B-lines, continuous	
Irregular visceral pleura, normal/abnormal	
Irregular visceral pleura, continuous	
Thickened parietal pleura, normal/abnormal	
Thickened parietal pleura, continuous	
Pulmonary diseases	
B-lines, normal/abnormal	
B-lines, continuous	
Irregular visceral pleura, normal/abnormal	
Irregular visceral pleura, continuous	
Thickened parietal pleura, normal/abnormal	
Thickened parietal pleura, continuous	
Pleural diseases	
B-lines, normal/abnormal	
B-lines, continuous	
Irregular visceral pleura, normal/abnormal	
Irregular visceral pleura, continuous	
Thickened parietal pleura, normal/abnormal	
Thickened parietal pleura, continuous	
Airway diseases	
B-lines, normal/abnormal	
B-lines, continuous	
Irregular visceral pleura, normal/abnormal	
Irregular visceral pleura, continuous	
Thickened parietal pleura, normal/abnormal	
Thickened parietal pleura, continuous	

TUS - Thoracic ultrasound; DOR – Diagnostic Odds ratio; 95% CI – 95% confidence interval

Mock-up Appendix 2b. Worst-case scenario. Univariate regression analyses comparing diagnostic odds ratio of different thoracic ultrasound measures modalities when identifying interstitial lung diseases, pulmonary diseases, pleural diseases, and airway diseases in systemic lupus erythematosus.

TUS modality	DOR (95% CI)
Interstitial lung diseases	
B-lines, normal/abnormal	
B-lines, continuous	
Irregular visceral pleura, normal/abnormal	
Irregular visceral pleura, continuous	
Thickened parietal pleura, normal/abnormal	
Thickened parietal pleura, continuous	
Pulmonary diseases	
B-lines, normal/abnormal	
B-lines, continuous	
Irregular visceral pleura, normal/abnormal	
Irregular visceral pleura, continuous	
Thickened parietal pleura, normal/abnormal	
Thickened parietal pleura, continuous	
Pleural diseases	
B-lines, normal/abnormal	
B-lines, continuous	
Irregular visceral pleura, normal/abnormal	
Irregular visceral pleura, continuous	
Thickened parietal pleura, normal/abnormal	
Thickened parietal pleura, continuous	
Airway diseases	
B-lines, normal/abnormal	
B-lines, continuous	
Irregular visceral pleura, normal/abnormal	
Irregular visceral pleura, continuous	
Thickened parietal pleura, normal/abnormal	
Thickened parietal pleura, continuous	

TUS - Thoracic ultrasound; DOR – Diagnostic Odds ratio; 95% CI – 95% confidence interval

Mock-up Appendix 3. Only for patients with BMI lower than 30%. Univariate regression analyses comparing diagnostic odds ratio of different thoracic ultrasound measures modalities when identifying interstitial lung diseases, pulmonary diseases, pleural diseases, and airway diseases in systemic lupus erythematosus.

TUS modality	DOR (95% CI)
Interstitial lung diseases	
B-lines, normal/abnormal	
B-lines, continuous	
Irregular visceral pleura, normal/abnormal	
Irregular visceral pleura, continuous	
Thickened parietal pleura, normal/abnormal	
Thickened parietal pleura, continuous	
Pulmonary diseases	
B-lines, normal/abnormal	
B-lines, continuous	
Irregular visceral pleura, normal/abnormal	
Irregular visceral pleura, continuous	
Thickened parietal pleura, normal/abnormal	
Thickened parietal pleura, continuous	
Pleural diseases	
B-lines, normal/abnormal	
B-lines, continuous	
Irregular visceral pleura, normal/abnormal	
Irregular visceral pleura, continuous	
Thickened parietal pleura, normal/abnormal	
Thickened parietal pleura, continuous	
Airway diseases	
B-lines, normal/abnormal	
B-lines, continuous	
Irregular visceral pleura, normal/abnormal	
Irregular visceral pleura, continuous	
Thickened parietal pleura, normal/abnormal	
Thickened parietal pleura, continuous	

TUS - Thoracic ultrasound; DOR – Diagnostic Odds ratio; 95% CI – 95% confidence interval
