# Supplementary material for: Thoracic ultrasound finding interstitial lung disease in SLE: a diagnostic test accuracy study
Source: Rheumatol Adv Pract. 2026 Oct 14;10(4):rkag126. doi: 10.1093/rap/rkag126 (PMC13713183; doi:10.1093/rap/rkag126)
Supplement: Supplementary file 1 [file rkag126_supplementary_data.zip › Supplementary Data S3.pdf]

Supplementary Data S3. Prevalence of ILD among 185 patients with SLE.

Types of PD	Diagnoses of PD	Prevalence, no. (%)
PD		109 (59%)
ILD		22 (12%)
	UIP	1 (0.54%)
	Probable UIP	4 (2.2%)
	Indeterminate for UIP	5 (2.7%)
	Fibrotic NSIP	1 (0.54%)
	Fibrotic HP	2 (1.1%)
	Non-fibrotic HP	1 (0.54%)
	LIP	3 (1.6%)
	OP	1 (0.54%)
	Other*	6 (3.2%)

The percentage is of the total study population. PD – pulmonary diseases; ILD – interstitial lung diseases; UIP – usual interstitial pneumonia; NSIP – non-specific interstitial pneumonia; HP – hypersensitivity pneumonitis; LIP – lymphoid interstitial pneumonia; OP – organising pneumonia.
