# Supplementary material for: Thoracic ultrasound finding interstitial lung disease in SLE: a diagnostic test accuracy study
Source: Rheumatol Adv Pract. 2026 Oct 14;10(4):rkag126. doi: 10.1093/rap/rkag126 (PMC13713183; doi:10.1093/rap/rkag126)
Supplement: Supplementary file 1 [file rkag126_supplementary_data.zip › Supplementary Data S4.pdf]

Supplementary Data S4, 2 x 2 contingency tables.

Interstitial lung diseases, ILD

5 ≥ b-lines

	ILD Positive 22	ILD Negative 163
TUS Positive 72	16	56
TUS Negative 113	6	107

8 ≥ B-lines

	ILD Positive 22	ILD Negative 163
TUS Positive 43	14	29
TUS Negative 142	8	134

9 ≥ B-lines

	ILD Positive 22	ILD Negative 163
TUS Positive 38	14	24
TUS Negative 147	8	139

10 ≥ B-lines

	ILD Positive 22	ILD Negative 163
TUS Positive 37	14	23
TUS Negative 148	8	140

11 ≥ B-lines

	ILD Positive 22	ILD Negative 163
TUS Positive 32	12	20
TUS Negative 153	10	143

12 ≥ B-lines

	ILD Positive 22	ILD Negative 163
TUS Positive 29	11	18
TUS Negative 156	11	145

15 ≥ B-lines

	ILD Positive 22	ILD Negative 163
TUS Positive 21	11	10

TUS Negative 164	11	153

20 ≥ B-lines

	ILD Positive 22	ILD Negative 163
TUS Positive 11	7	4
TUS Negative 174	15	159

25 ≥ B-lines

	ILD Positive 22	ILD Negative 163
TUS Positive 7	5	2
TUS Negative 178	17	161

Irregular visceral pleura ≥ 2 zones

	ILD Positive 22	ILD Negative 163
TUS Positive 42	11	31
TUS Negative 143	11	132

Thickened parietal pleura ≥ 1 zones

	ILD Positive 22	ILD Negative 163
TUS Positive 21	6	15
TUS Negative 164	16	148

No ILD without B-lines – cystic ILD, sarcoidosis, and organizing pneumonia

Sensitivity B-lines and BMI 30, threshold ≥ 10 B-lines

	+ ILD (19)	- ILD (129)
TUS positive (32)	13	19
TUS Negative (116)	6	110

Sensitivity B-lines and threshold ≥ 10 B-lines and No ILD without B-lines

	+ ILD (17)	- ILD (163)
TUS positive (36)	13	23
TUS Negative (144)	4	140

Sensitivity B-lines and BMI 30, threshold ≥ 10 B-lines and No ILD without B-lines

	+ ILD (14)	- ILD (129)
TUS positive (30)	12	19
TUS Negative (113)	2	110

Sensitivity B-lines ≥ 10 and visceral abnormalities ≥ 1

	+ ILD (22)	- ILD (163)
TUS positive (88)	16	72
TUS Negative (97)	6	91

Sensitivity B-lines ≥ 10 and visceral abnormalities ≥ 1 and No ILD without B-lines

	+ ILD (17)	- ILD (163)
TUS positive (86)	14	72
TUS Negative (94)	3	91

Sensitivity B-lines ≥ 10 and visceral abnormalities ≥ 1 and No BMI over 30

	+ ILD (19)	- ILD (129)
TUS positive (69)	15	54
TUS Negative (79)	4	75

Sensitivity B-lines ≥ 10 and visceral abnormalities ≥ 1 and No BMI over 30 and No ILD without B-lines

	+ ILD (14)	- ILD (129)
TUS positive (67)	13	54
TUS Negative (76)	1	75

ILD with patients BMI over 30

	+ ILD (3)	- ILD (34)
TUS positive (5)	1	4
TUS Negative (30)	2	30
