# Supplementary material for: Thoracic ultrasound finding interstitial lung disease in SLE: a diagnostic test accuracy study
Source: Rheumatol Adv Pract. 2026 Oct 14;10(4):rkag126. doi: 10.1093/rap/rkag126 (PMC13713183; doi:10.1093/rap/rkag126)
Supplement: Supplementary file 1 [file rkag126_supplementary_data.zip › Supplementary Data S5-S10.pdf]

Supplementary Data S5-S10

Supplementary Data S5. Summary of the diagnostic performance of B-lines in identification of ILD in 185 patients with SLE.

Threshold, number of b-lines	DOR, (95%CI)	Sensitivity, (95%CI)	Specificity, (95%CI)	PPV, (95%CI)	NPV, (95%CI)	Youden Index
ILD						
≥5	5.10 (1.76 to 17.7)	72.7% (49.8% to 89.3%)	65.6% (57.8% to 72.9%)	22.2% (13.3% to 33.6%)	94.7% (88.8% to 98.0%)	0.38
≥8	8.09 (2.81 to 24.1)	63.6% (40.7% to 82.8%)	82.2% (75.5% to 87.7%)	32.6% (19.1% to 48.5%)	94.4% (89.2% to 97.5%)	0.46
≥9	10.1 (3.47 to 30.6)	63.6% (40.7% to 82.8%)	85.3% (78.9% to 90.3%)	36.8% (21.8% to 54.0%)	94.6% (89.6% to 97.6%)	0.49
≥10 * optimal threshold	10.7 (3.67 to 32.3)	63.6% (40.7% to 82.8%)	85.9% (79.6% to 90.8)	37.8% (22.5% to 55.2%)	96.6% (89.5% to 97.6%)	0.50
≥11	8.58 (2.92 to 25.0)	54.5% (32.2% to 75.6%)	87.7% (81.7% to 92.3%)	37.5% (21.1% to 56.3%)	93.5% (88.3% to 96.8%)	0.42
≥12	8.06 (2.70 to 23.6)	50.0% (28.2% to 71.8%)	89.0% (83.1% to 93.3%)	37.9% (20.7% to 57.7%)	92.9% (87.7% to 96.4%)	0.39
≥15	15.3 (4.65 to 49.6)	50.0% (28.2% to 71.8%)	93.9% (89.0% to 97.0%)	52.4% (29.8% to 74.3%)	93.3% (88.3% to 96.6%)	0.44
≥20	18.6 (4.04 to 93.6)	31.8% (13.9% to 54.9%)	97.5% (93.8% to 99.3%)	63.6% (30.8% to 89.1%)	91.4% (86.2% to 95.1%)	0.29
≥25	23.7 (3.42 to 257)	22.7% (7.8% to 45.4%)	98.8% (95.6% to 99.9%)	71.4% (29.0 % to 96.3%)	90.4 (85.1% to 94.3%)	0.22

* - optimal threshold defined by Youden Index. DOR – diagnostic odds ratio; 95%CI – 95% confidence interval; PPV – positive predictive value; NPV – negative predictive value; ILD – Interstitial lung diseases.

Supplementary Data S6. Diagnostic performance of different TUS modalities when identifying ILD in 185 patients with SLE.

Thoracic ultrasound modality	DOR (95%CI)	AUC (95%CI)
	ILD	
B-lines, normal/abnormal ≥10†	10.7 (3.67 to 32.3)***	NA
B-lines, continuous	1.148 (1.079 to 1.221)***	0.81 (0.71 to 0.91)
Irregular visceral pleura, normal/abnormal ≥2†	4.26 (1.51 to 11.8)**	NA
Irregular visceral pleura, continuous	1.540 (1.214 to 1.952)**	0.67 (0.53 to 0.80)
Thickened parietal pleura, normal/abnormal ≥1†	3.70 (1.02 to 11.9)*	NA
Thickened parietal pleura, continuous	1.776 (1.233 to 2.558)**	0.62 (0.50° to 0.75)

* P-value < 0.01, ** P-value < 0.005, *** P-value < 0.001, † - defined by Youden index, ° - 0.4978. TUS - thoracic ultrasound; DOR – diagnostic odds ratio; 95%CI – 95% confidence interval; AUC – area under the curve; NA – not applicable.

Supplementary Data S7. Participants with ILD and less than 10 B-lines.

Participant Number	No of B-lines	BMI (kg/m²)	Type of ILD	FVC / DLCO (% of expected)	Nr of zones with abnormal visceral / thickened parietal pleura
#1	3	25	DCLD*	114 / 81	0 / 0
#2	3	26	DCLD*	107 / 65	0 / 0
#3	7	28	OP*	71 / 37	2 / 1
#4	2	27	DCLD*	81 / 76	0 / 0
#5	4	30.1**	HP	62 / 56	0 / 1
#6	3	45**	ILA	90 / 96	0 / 0
#7	1	29.7	NSIP	97 / 80	0 / 1
#8	7	20	UIP	77 / 56	2 / 4

* Types of ILD that often do not relate to B-lines. ** - BMI ≥ 30. No – number; BMI – body mass index; ILD – interstitial lung diseases; FVC – forced vital capacity; DLCO – diffusing capacity for the lung for carbon monoxide; DCLD – diffuse cystic lung disease; OP – organising pneumonia; HP – hypersensitivity pneumonia; ILA – interstitial lung abnormalities; NSIP – non-specific interstitial pneumonia; UIP - usual interstitial pneumonia.

Supplementary Data S8. Participants without ILD but 10 B-lines or more.

#	# B-lines	Pulmonary diseases	Cardiovascular diseases*	Other relevant
1	13	Obliterative bronchiolitis, Bronchiectasis	Cardiomegaly	None
2	19	Bronchiectasis	None	Breast cancer, treated with local radiation
3	12	None	None	None
4	10	Pleuraparenchymal fibrosis, Obliterative bronchiolitis	Pericarditis, Prior pulmonary embolism	None
5	17	Thickened airways	None	None
6	14	Pleuraparenchymal fibrosis, Emphysema	Ischemic heart disease, Heart failure	None
7	16	Active pleuritis, Emphysema, Bronchiectasis, Thickened airways	Ischemic heart disease	None
8	13	Pleuraparenchymal fibrosis, Bronchiectasis	None	None
9	10	Pleuraparenchymal fibrosis, Thickened airways	None	None
10	25	Pleuraparenchymal fibrosis, Bronchiectasis	Prior pulmonary embolism	None
11	21	Active pleuritis with effusions	None	None
12	22	Bronchiectasis, Thickened airways, Mucus plugging	None	None
13	13	Bronchiectasis	Valvular heart disease	None
14	13	Emphysema	None	Repeated RIT
15	17	Thickened airways	None	None
16	11	Pleuraparenchymal fibrosis, Emphysema, COPD, Bronchictasis, Thickened airways	None	None
17	15	Apical lung cysts, Pleuraparenchymal fibrosis, Bronchiectasis	None	None
18	12	Active pleuritis	Prior pericarditis	None
19	18	Pulmonal hypertension Pleuraparenchymal fibrosis, Bronchiectasis,	Valvular heart disease	None
20	13	Airtrapping	Prior pericarditis	None
21	11	Micro nodulus, Bronchiectasis	Valvular disease, Left sided heart failure, Pulmonal hypertension,	None
22	29	Left lover lobe lobectomy, Emphysema COPD	None	Tracheomalacia
23	10	None	None	None

*based on review of medical records. COPD – Chronic obstructive pulmonary disease; RIT – Respiratory tract infection.

Supplementary Data S9. Sensitivity analyses investigating the performance of B-lines.

TUS modality	Sensitivity (95%CI) / Specificity (95%CI)	DOR (95%CI)	AUC (95%CI)
Without BMI ≥30			
Normal/abnormal ≤ 10 B-lines*, n=148	68% (43% to 87%) / 85% (78% to 91%)	12.5 (3.79 to 44.4)	NA
B-lines, continuous	NA	1.15 (1.08 to 1.24)	0.82 (0.70 to 0.93)
With BMI ≥30			
Normal/abnormal ≤ 10 B-lines*, n=37	33% (0.84% to 91%) / 88% (73% to 97%)	3.75 (0.05 to 84.8)	NA
B-lines, continuous	NA	1.11 (0.99 to 1.26)	0.75 (0.47 to 1.00)
Without certain ILD†			
Normal/abnormal ≤ 10 B-lines*, n=180	76% (50% to 93%) 86% (80% to 91%)	19.8 (5.36 to 88.2)	NA
B-lines, continuous	NA	1.18 (1.10 to 1.26)	0.86 (0.75 to 0.97)
Without BMI ≥*30 and certain ILD†*			
Normal/abnormal ≤ 10 B-lines*, n=143	86% (57% to 98%) / 85% (78% to 91%)	34.7 (6.69 to 330)	NA
B-lines, continuous	NA	1.18 (1.09 to 1.28)	0.89 (0.78 to 1.00)
Combining B-lines ≥10* and visceral pleural abnormalities ≥1*			
Normal/abnormal, n=185	73% (50% to 89%) / 56% (48% to 64%)	3.37 (1.17 to 11.0)	NA
Combining B-lines ≥ 10* and visceral pleural abnormalities ≥1* and *without BMI ≥30*			
Normal/abnormal, n=148	79% (54% to 94%) / 58% (49% to 67%)	5.21 (1.33 to 22.5)	NA
Combining B-lines ≥10* and visceral pleural abnormalities ≥1* *without certain ILD†*			
Normal/abnormal, n=180	82% (57% to 96%) / 56% (48% to 64%)	5.90 (1.55 to 32.9)	NA
Combining B-lines ≥10* and visceral pleural abnormalities ≥1* *without BMI ≥30 and certain ILD†*			
Normal/abnormal, n=143	93% (66% to 100%) / 58% (49% to 67%)	18.1 (2.53 to 777)	NA

*=optimal threshold according to Youden Index; † - Interstitial lung diseases that often do not give rise to B-lines (Cystic, Sarcoidosis, and Organising Pneumonitis); 95%CI – 95% confidence interval; DOR – diagnostic odds ratio; BMI – body mass index; NA – not applicable; ILD – interstitial lung diseases.

Supplementary Data S10. Outcome measures for TUS detecting ILD in SLE with cut-off ≥10 B-lines, excluding participants with BMI 30 kg/m^2 and/OR with certain ILD

Outcome measure	Result
Diagnostic odds ratio (95%CI)	34.7 (6.7 to 330)
Sensitivity (95%CI)	86% (57% to 98%)
Specificity (95%CI)	85% (78% to 91%)
Positive predictive value (95%CI)	39% (22% to 58%)
Negative predictive value (95%CI)	98% (94% to 100%)
Positive likelihood ratio (95%CI)	5.8 (3.7 to 9.3)
Negative likelihood ratio (95%CI)	0.17 (0.05 to 0.61)
Youden index	0.71

95%CI – 95% confidence interval
